# Cell Therapy Attenuates Cardiac Dysfunction Post Myocardial Infarction: Effect of Timing, Routes of Injection and a Fibrin Scaffold

**DOI:** 10.1371/journal.pone.0006005

**Published:** 2009-06-23

**Authors:** Juliana S. Nakamuta, Maria E. Danoviz, Fabio L. N. Marques, Leonardo dos Santos, Claudia Becker, Giovana A. Gonçalves, Paula F. Vassallo, Isolmar T. Schettert, Paulo J. F. Tucci, Jose E. Krieger

**Affiliations:** 1 Heart Institute (InCor), University of São Paulo Medical School, São Paulo, São Paulo, Brazil; 2 Radiopharmacy Laboratory, Nuclear Medicine Center, University of São Paulo Medical School, São Paulo, São Paulo, Brazil; 3 Cardiac Physiology and Pathophysiology Laboratory, Cardiology Division, Federal University of Sao Paulo, São Paulo, São Paulo, Brazil; Instituto de Química, Universidade de São Paulo, Brazil

## Abstract

**Background:**

Cell therapy approaches for biologic cardiac repair hold great promises, although basic fundamental issues remain poorly understood. In the present study we examined the effects of timing and routes of administration of bone marrow cells (BMC) post-myocardial infarction (MI) and the efficacy of an injectable biopolymer scaffold to improve cardiac cell retention and function.

**Methodology/Principal Findings:**

^99m^Tc-labeled BMC (6×10^6^ cells) were injected by 4 different routes in adult rats: intravenous (IV), left ventricular cavity (LV), left ventricular cavity with temporal aorta occlusion (LV^+^) to mimic coronary injection, and intramyocardial (IM). The injections were performed 1, 2, 3, or 7 days post-MI and cell retention was estimated by γ-emission counting of the organs excised 24 hs after cell injection. IM injection improved cell retention and attenuated cardiac dysfunction, whereas IV, LV or LV* routes were somewhat inefficient (<1%). Cardiac BMC retention was not influenced by timing except for the IM injection that showed greater cell retention at 7 (16%) vs. 1, 2 or 3 (average of 7%) days post-MI. Cardiac cell retention was further improved by an injectable fibrin scaffold at day 3 post-MI (17 vs. 7%), even though morphometric and function parameters evaluated 4 weeks later displayed similar improvements.

**Conclusions/Significance:**

These results show that cells injected post-MI display comparable tissue distribution profile regardless of the route of injection and that there is no time effect for cardiac cell accumulation for injections performed 1 to 3 days post-MI. As expected the IM injection is the most efficient for cardiac cell retention, it can be further improved by co-injection with a fibrin scaffold and it significantly attenuates cardiac dysfunction evaluated 4 weeks post myocardial infarction. These pharmacokinetic data obtained under similar experimental conditions are essential for further development of these novel approaches.

## Introduction

Transplantation of stem and progenitor cells is emerging as a promising therapeutic option for repair of ischemic and infarcted myocardium [1]–[3]. Nevertheless, the implementation of this novel approach in a clinical setting requires the understanding of a number of key aspects that remain poorly understood. Among the main issues, the optimal timing for therapy and the most appropriate cell delivery route might be of particular importance in order to maximize cell transplantation efficiency.

Studies investigating the kinetics of cytokines production and mobilization of stem cells to the injured myocardium provide evidence that these processes occur within a limited time window after infarction [4]–[6]. This provides a rational for identification of the ideal timing for cell transplantation. Moreover, recent evidence suggests that the combination of cells with biopolymers such as fibrin, collagen and matrigel can improve cell survival, angiogenesis and cardiac function [7]–[9].

Different routes for cell administration have been proposed to deliver cells including transepicardic [10]–[14], systemic [15]–[17] and intracoronary balloon catheter-mediated cell delivery [18]–[21], however, comparative studies designed to evaluate both their efficiencies and the effect of timing are scarce. The optimal timing for therapy may vary depending on the route used to administer the cells and factors associated to cell *in situ* retention at the damaged cardiac tissue may be essential for the intramuscular (IM) injection, whereas cell recruitment should be equally important for intravenous injection. Consequently, the optimal time to transplant the cells should be assessed within the context of the delivery modality in order to optimize the efficiency of the predominant underlying mechanism(s) related to each route.

In the present study, we examined the effect of timing and routes of administration of ^99m^Tc-labeled bone marrow cells (BMCs) on cardiac cell retention, as well as the efficacy of a biopolymer used as vehicle to improve retention and cardiac function in rats submitted to experimental myocardium infarction (MI).

## Results

### Labeling efficiency and stability

BMCs showed an average labeling efficiency of 14.9±3.5%, which means that approximately 15% of the total radioactivity labeled uniformly the cell pool, resulting in 1.98 MBq per 10^6^ BMCs. Moreover, the ^99m^Tc radioactivity detected in BMCs and in the supernatant revealed that only 33.0±2.5% of the radioactivity initially incorporated remained within the cells 24 hours after labeling. The absence of significant deterioration of cell viability (80% for labeled vs. 87.5% for unlabeled BMCs, *P*>0.05), as indicated by the Trypan blue dye exclusion test, suggests that the radioactivity detected in the supernatant is secondary to ^99m^Tc leakage from labeled BMCs rather than cell death. Therefore, to determine the transplanted cell accumulation, heart radioactivity values were corrected according to the rate of ^99m^Tc leakage from BMCs *in vitro* (33.0%) assuming an equivalent rate of leakage *in vivo*.

### Cardiac cell accumulation according to timing of therapy

**Figure 1:**
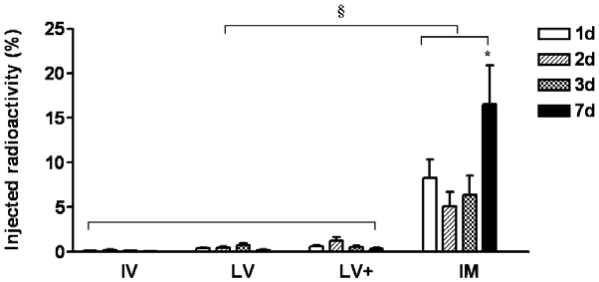
Effect of timing of injection by different routes on cardiac ^99m^Tc-labeled BMC retention. Cells were injected 1, 2, 3 or 7 days after myocardial infarction by different routes: intravenous (IV) (N = 20), left ventricular cavity (LV) (N = 17), “intracoronary” (LV^+^) (N = 17), and intramyocardial (IM) (N = 21). Data is presented a percentage means (±SE). * (*P*<0.01) indicates statistical difference compared to IM 1, 2 and 3d and § (*P*<0.001) indicates statistical difference compared to IV, LV and LV^+^ routes.

To determine the best moment to administer cells post-MI, the radiolabeled BMCs were delivered through 4 different routes at 1, 2, 3 or 7 days post-infarction. Cardiac BMCs retention was not influenced by timing of injection in IV, LV and LV^+^ routes. For the IM injection, however, there was greater cell retention in the 7 days post-MI group compared to BMCs injected after 1, 2 or 3 days after MI (Table S1 and Figure 1).

### Cardiac cell accumulation and routes of administration

Regardless of timing of injection, the IM route resulted in greater cell retention ranging from approximately 7% when cells were injected 1, 2, or 3 days post-MI and about 16% on 7 days post-MI group. In contrast there was very small retention of less than 1% for all the remaining routes despite the timing of injection (Table S1 and Figure 1).

To assess whether the radioactivity retention associated with the IM group reflected BMC accumulation, instead of a combination of BMCs with leaked tracer remaining in the tissue, radiolabeled BMCs were incubated for 24 hours, centrifuged and then the supernatant injected by the IM route. The amount of ^99m^Tc metabolites retained in the heart was 12.5 times lower than the values observed with the labeled injected cells (0.21±0.05 vs. 2.62±0.39, respectively) indicating that the contribution of free ^99m^Tc to total myocardium radioactivity can indeed be disregarded.

### Body distribution of cells according to routes of administration

**Figure 2:**
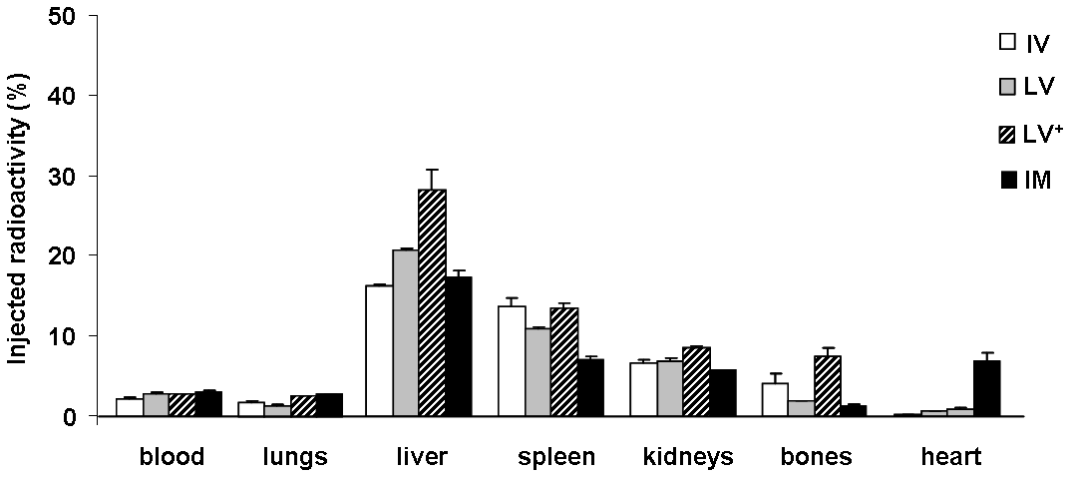
Effect of different routes of injection of ^99m^Tc-labeled BMC on radioactivity tissue distribution profile. Cells were injected post-MI via intravenous (IV) (N = 20), left ventricular cavity (LV) (N = 17), “intracoronary” (LV^+^) (N = 17), and intramyocardial (IM) (N = 21) routes. Data are expressed as percentage means (±SE).

Interestingly, the biodistribution pattern of radiolabeled BMCs, assessed 24 hours following each injection, was similar for all delivery routes (Figure 2). Approximately 50% of the injected radioactivity was found in the main harvested organs. High radioactivity values were detected in liver, spleen and kidneys, with markedly lower values in lungs and bones.

### Use of a biopolymer scaffold as a strategy to improve cardiac cell retention

**Figure 3:**
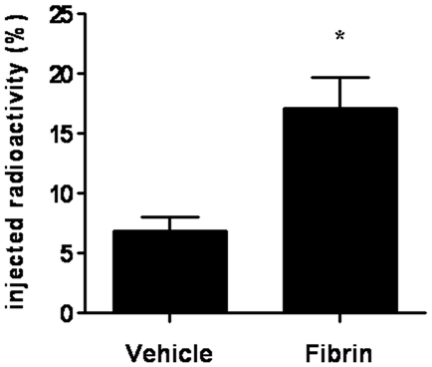
Effect of intramyocardial co-injection of a fibrin scaffold on cardiac cell retention post-myocardial infarction. ^99m^Tc-labeled BMC were injected post-myocardial infarction directly into the myocardium in vehicle (DMEM, N = 22) or in a fibrin scaffold (Fibrin, N = 15). Cell retention was assessed 24 hours later by the level of radioactivity in the heart shown as percentage means (±SE). * denotes statistical significance for *P*<0.01.

Given that the first 3 days post-MI are critical to halt cardiac damage and to improve cardiac repair since key molecules associated with homing, adhesion, proliferation, and cell survival are mainly available during this period and that in the best scenario, under the IM injection, only approximately 7% of the injected radioactivity remain in the myocardium, an autologous fibrin polymer was tested as vehicle for BMC injection to improve cell retention. The IM injection of BMCs using the fibrin polymer increased significantly cell retention compared with standard vehicle injected 3 days post-MI (17.12±2.61 vs. 6.84±1.17%, *P* = 0.0005) (Figure 3).

To examine the possibility that the increased radioactivity retention observed in the fibrin group was secondary to trapping of free tracer in the polymer matrix, 10 µL of supernatant containing ^99m^Tc leaked from BMCs were added to the fibrinogen solution before adding thrombin *in vitro*. After 24 hours of incubation in 500 µL of DMEM followed by two washes in PBS, only 1.29±0.07% of the total radioactivity remained bound to the fibrin polymer indicating that free ^99m^Tc is washed out and does not remain trapped into the fibrin polymer matrix.

To further confirm the efficacy of this strategy, we tested the injectable fibrin scaffold with other more homogeneous cell types including rat cardiac fibroblasts expressing β-galactosidase and rat adipose stem cells (see supplementary Methods S1). Twenty-four hours after injection of cardiac fibroblasts there was higher cell retention in the fibrin compared to the control group (supplementary Figure S1). Furthermore, adipose mesenchymal stem cells injected with fibrin survived and remained in the myocardium for at least 30 days (supplementary Figure S2).

### Effect of increased BMC accumulation on cardiac morphometry and performance

To examine if greater BMC cardiac retention translated into improved cardiac repair, morphometric and functional analyses were performed in 5 groups of rats administered with BMCs IV or IM and for the former in the presence or absence of fibrin 4 weeks after myocardial infarction (Table 1).

**Table 1:** Experimental design of functional study.

Group	MI	Content	Route
SHAM (N = 5)	−	−	−
M - IM (N = 4)	+	culture medium	intramyocardial (IM)
F - IM (N = 4)	+	fibrin	intramyocardial (IM)
BMC - IV (N = 5)	+	BMC	intravenous (IV)
BMC - IM (N = 4)	+	BMC	intramyocardial (IM)
BMC+F - IM (N = 4)	+	BMC+fibrin (1×10^6^)	intramyocardial (IM)

*N* indicates the number of animals in each group. + and − indicates the presence or absence of myocardial infarction (MI).

### Infarct size, LV cavity perimeter and scar thickness

**Figure 4:**
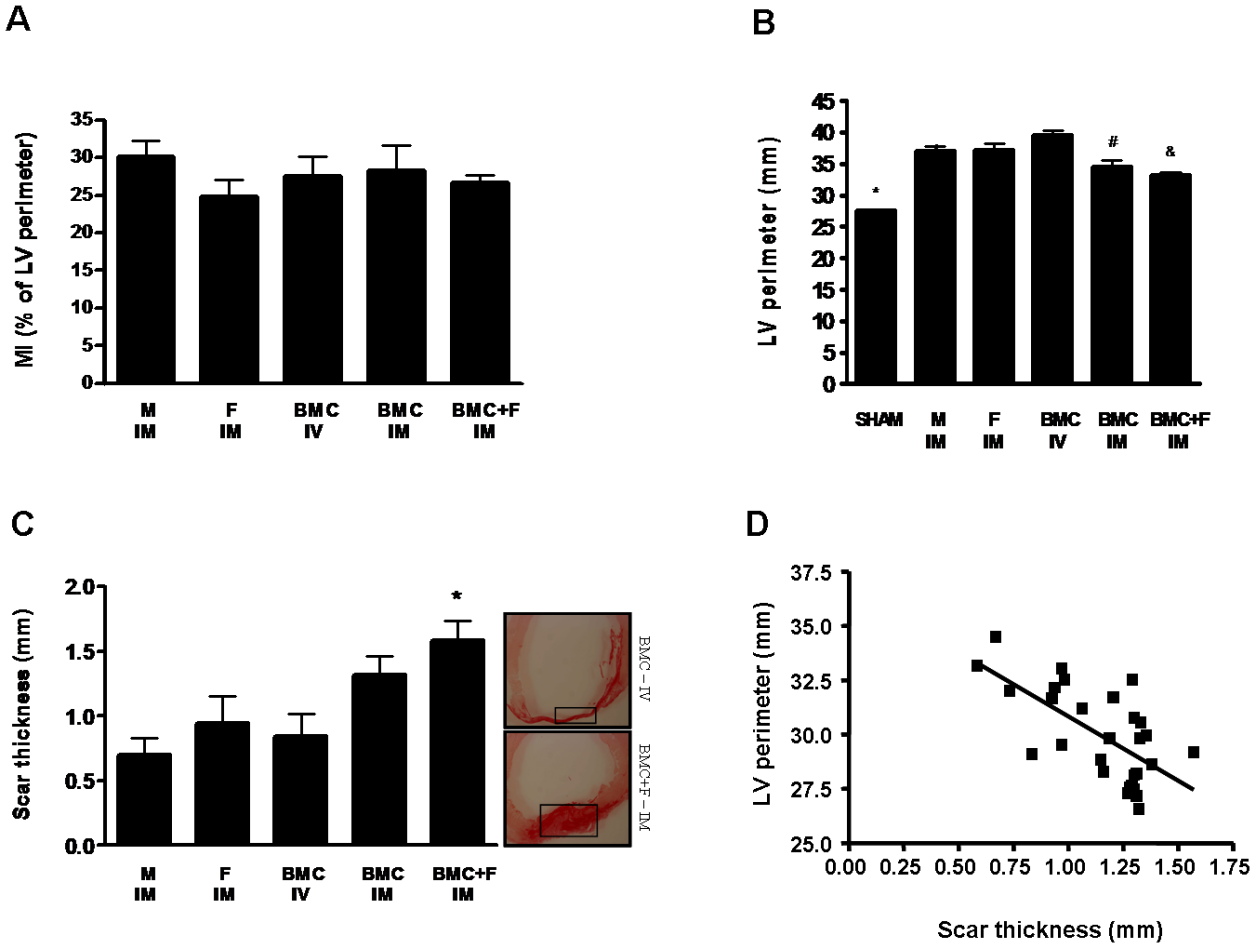
Effect of intramyocardial co-injection of fibrin scaffold 3 days post-MI on cardiac morphometry. (A) Myocardial infarction estimated as a percentage of left ventricle (LV) perimeter with a scar (mean±SE). (B) Left ventricle (LV) perimeter in millimeters (mean±SE); * indicates statistical difference compared to all groups for *P*<0.001, ^#^ indicates statistical difference compared to BMC-IV group for *P*<0.001 and ^&^ indicates statistical difference compared to F-IM and BMC-IV for *P*<0.01. (C) Scar thickness in millimeters (mean±SE); * indicates statistical difference compared to M-IM and BMC-IV groups for *P*<0.05. (D) Linear regression of scar thickness as a function a LV perimeter (Pearson's r = −0.65; slope = −5.91±1.31, *P*<0.001).

Histological analyses revealed no significant differences in percentage of left ventricle perimeter occupied by fibrosis between the 5 groups (31.21±1.99 vs. 24.76±2.27 vs. 27.51±2.67 vs. 28.25±3.41 vs. 26.66±1.04% for M-IM, F-IM, BMC-IV, BMC-IM, and BMC+F-IM, respectively, *P* = 0.6384) (Figure 4A).

The LV perimeter increased in all MI groups compared to the sham operated animals (27.5±0.18 vs. 36,98±0,78; 37,23±0,91, 39,48±0,77; 34.53±0.96; and 33,20±0.38 mm for Sham vs. M-IM, F-IM, BMC-IV, BMC-IM, and BMC+F-IM, respectively, *P*<0.001), but was significantly reduced in the groups that received BMC injected IM, with or without the fibrin scaffold matrix, compared to the other experimental groups (Figure 4B). Similarly, the scar thickness tended to be thicker in the animals injected with BMC IM, but reached significant values only in the group injected with the fibrin scaffold (1.58±0.15 vs. 0.70±0.13, 0.94±0.20 0.85±0,16, 1.3±0.14 for BMC+F-IM vs. M-IM, F-IM, BMC-IV, and BMC-IM, respectively, p<0.05) (Figure 4C).

Interestingly, the linear regression model from data of scar thickness and LV perimeter shows a significant inverse correlation between LV cavity dilatation and scar thickness (Pearson's r = −0.65; slope = −5.91±1.31, *P*<0.001) (Figure 4D).

### Hemodynamic assessment of ventricular function

We next examined whether the improvement in cardiac remodeling associated with BMC administered IM, with or without fibrin scaffold, translated into functional benefits. As a show in table 2, the indices of ventricular function under basal conditions were generally worse in M-IM and F-IM groups compared to sham group. LVEDP was increased in M-IM group and the LVSP diminished in M-IM and BMC-IV groups. Moreover, low values of +dP/dt_max_ were observed in M-IM, F-IM and BMC-IV compared to sham animals. The other hemodynamic parameters exhibited similar profile in all experimental groups.

**Table 2:** Hemodynamics parameters under basal conditions of experiment.

	Sham	M-IM	F-IM	BMC-IV	BMC-IM	BMC+F-IM	*P* value
	n = 5	n = 5	n = 4	n = 6	n = 6	n = 6	
LVEDP (mmHg)	3.78±0.91*	11.18±1.95	10.50±2.75	7.40±1.20	5.50±0.86	6.00±1.41	0.02
LVSP (mmHg)	121.50±2.63* #	104.10±2.81	106.80±4.40	102.60±4.35	115.50±3.88	108.80±4.78	0.009
+dP/dt_max_ (mmHg.s^−1^)	9.158±491.30* & #	5.976±308.70	7.062±345.60	6.582±329.70	8.437±786.10* &	7.635±272.90	0.0002
−dP/dt_max_ (mmHg.s^−1^)	−5.324±382.50	−4.405±281.30	−4.619±204.10	−4.550±437.00	−4.974±217.90	−4.651±226.70	0.30
HR (bpm)	329.10±22.57	309.40±13.64	372.30±25.92	328.00±10.24	339.00±14.08	341.80±8.26	0.21
CI (mL.min^−1^/kg)	143.70±15.99	132±7.86	162.80±14.19	121.60±7.95	139.80±7.52	134.30±12.74	0.24
SVI (mL.beat^−1^/kg)	0.51±0.07	0.60±0.05	0.48±0.07	0.37±0.03*	0.41±0.01	0.39±0.03	0.03
SWI (gm.min^−1^/kg)	0.68±0.08	0.64±0.05	0.67±0.07	0.48±0.05	0.61±0.01	0.54±0.04	0.20

Left ventricular end diastolic pressure (LVEDP), left ventricular systolic pressure (LVSP), rate of LV pressure rise (dP/dt), heart rate (HR), cardiac index (CI), stroke volume index (SVI) and stroke work index (SWI) are presented as mean±SE. P value indicates statistic analises of groups for each parameter.

* indicates statistically significant difference compared to M-IM group (LVEDP: *P*<0.05, LVSP: *P*<0.05; +dP/dtmax: *P*<0.001).

# indicates statistically significant difference compared to BMC-IV group (LVSP: *P*<0.05; +dP/dtmax: *P*<0.01) and & indicates statistically significant difference compared to F-IM group (+dP/dtmax: *P*<0.05).

More informative data to evaluate overall cardiac function, however, were obtained during the pharmacologic pressure stress with phenilefrine. The animals that received BMC by intramyocardial route exhibited a clear improvement in myocardial function especially considering the discrete basal hemodynamic and morphometric changes described before.

**Figure 5:**
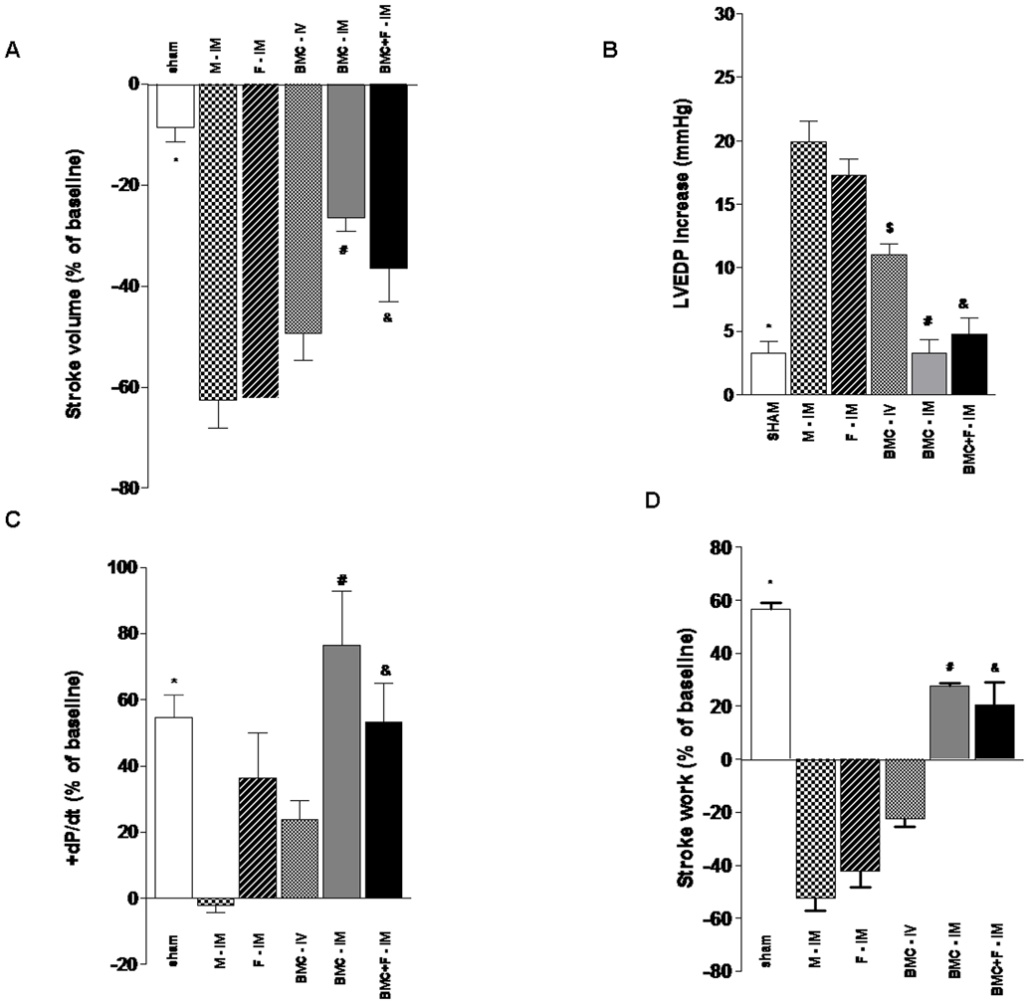
Effect of co-injection of fibrin scaffold on hemodynamics of left ventricle under pharmacologic pressure stress. Three days post-MI, animals were administered with medium intramuscular (M-IM), fibrin intramuscular (F-IM), BMC intravenous (BMC-IV), BMC intramuscular (BMC-IM), or BMC co-injected with fibrin intramuscular (BMC+F-IM) or animals were submitted to sham operation (Sham). (A) Change in stroke volume as a percentage change from baseline (mean±SE); * indicates statistical difference compared to all groups (M-IM, F-IM and BMC-IV: *P*<0.001, BMC: *P*<0.05 and BMC+F-IM: *P*<0.01), ^#^ indicates statistical difference compared to M-IM (*P*<0.001), F-IM (*P*<0.001) and BMC-IV (*P*<0.01) groups, ^&^ indicates statistical difference compared to M-IM (*P*<0.001), F-IM (*P*<0.001) and BMC-IV (*P*<0.05) groups. (B) Left ventricle end diastolic pressure (LVEDP) expressed in mmHg (mean±SE); * indicates statistical difference compared to M-IM, F-IM and BMC-IV groups for *P*<0.001, ^#^ indicates statistical difference compared to M-IM for *P*<0.001, F-IM (*P*<0.001) and BMC-IV (*P*<0.01) groups, ^&^ indicates statistical difference compared to M-IM for *P*<0.001, F-IM (*P*<0.001) and BMC-IV (*P*<0.05) groups, ^$^ indicates statistical difference compared to M-IM (*P*<0.001) and F-IM (*P*<0.05). (C) * *P*<0.01 indicates statistically significant difference compared to M-IM group, ^#^ indicates statistically significant difference compared to M-IM for *P*<0.001 and BMC-IV (*P*<0.05) groups, ^&^ indicates statistical difference compared to M-IM group for *P*<0.001. (D) * indicates statistical difference compared to all groups (M-IM, F-IM and BMC-IV: *P*<0.001, BMC: *P*<0.05 and BMC+F-IM: *P*<0.01), ^#^ indicates statistical difference compared to M-IM (*P*<0.001), F-IM (*P*<0.001) and BMC-IV (*P*<0.01) groups, ^&^ indicates statistical difference compared to M-IM (*P*<0.001), F-IM (*P*<0.001) and BMC-IV (*P*<0.05) groups.

**Figure 6:**
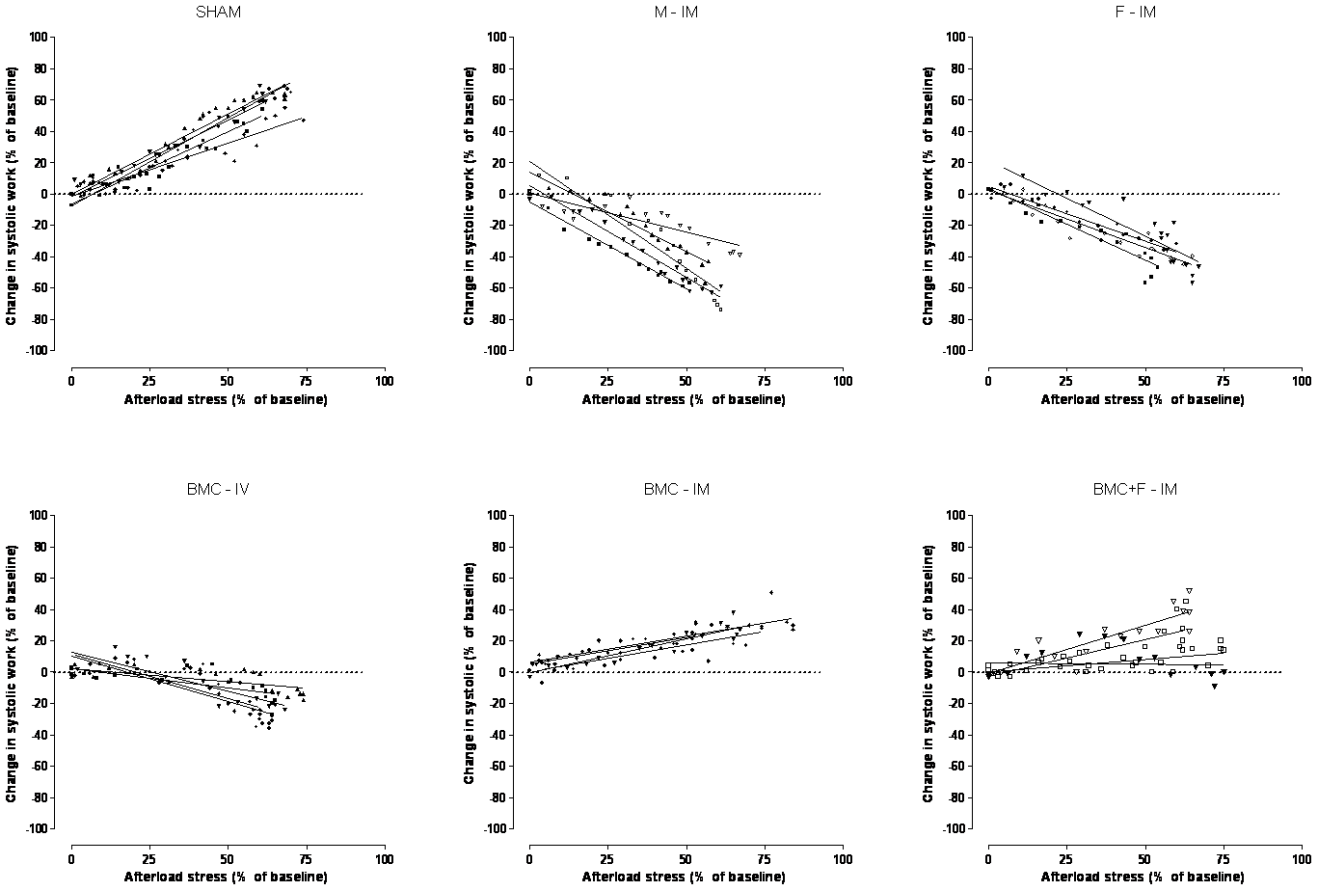
Linear regression curves of the stroke work as a function of increment in systolic pressure. Data are expressed as percentage change of baseline values for all experimental groups. See text for more details.

Stroke volume in response to pressure overload, depicted as % change, decreased in all infarcted groups, but it was better preserved in the groups receiving BMC IM either with or without the fibrin scaffold matrix compared to the other experimental groups (−4.55±2.19 vs. −62.50±5.50, −62.25±3.70, −49.40±5.26, −26.50±2.63, and −28.50±5.75% for sham vs. M-IM, F-IM, BMC-IV, BMC-IM, and BMC+F-IM, respectively, p<0.05) (Figure 5A). LVEDP increased only in the control experimental groups injected with medium and fibrin IM or BMC intravenously compared to sham animals or the IM BMC groups (3.26±0.97 vs. 19.90±1.66, 17.25±1.31, 11.00±0.89, 3.25±1.10, and 4.75±1.31 mmHg for Sham vs. M-IM, F-IM, BMC-IV, BMC-IM, and BMC+F-IM groups, respectively) (Figure 5B). The changes in +dP/dt_max_ were not as consistent as the other variables, but it can be noted that the index of contractility tended to reduce in the three control groups compared to sham, reaching significance only for the M-IM comparison, whereas it remained unchanged in the two BMC-IM groups (54.56+6.94 vs. −2.02±2.37, 36.25+13.64, 23.80±5.82, 76.50±16.36, 53.40±11.75% for Sham vs. M-IM, F-IM, BMC-IV, BMC-IM, and BMC+F-IM, respectively, *P*<0.01) (Figure 5c). Finally, the work generation under pressure overload, which represents a global index of cardiac function, showed clearly that intramyocardial injection of BMCs, with or without the fibrin scaffold, were the only treatments able to minimize the cardiac dysfunction associated to MI. A positive stroke work, although of smaller magnitude, was observed only for the MI groups receiving BMCs IM (56.70±2.32 vs. −52.54±4.66, −42.25±5.96, −22.60±8.70, 27.75±0.85, and 20.55±8.70% for Sham vs. M, F-IM, BMC-IV, BMC-IM, and BMC+F-IM groups, respectively) (Figure 5D). To further characterize the influence of the IM injection of BMCs, with or without fibrin, on stroke work, we plotted the individual linear regression curves for each group of the stroke work as a function of increment in systolic pressure (Figure 6). Positive correlations were noted only in Sham (Pearson's r = 0.95, mean slope  = 0.94±0.16), BMC-IM (Pearson's r = 0.85, mean slope  = 0.36±0.04) and BMC+F-M (Pearson's r = 0.76, mean slope  = 0.41±0.23) treated animals while negative correlations were observed in M-IM (Pearson's r = −0.93, mean slope  = −1.02±0.32), F-IM (Pearson's r = −0.93, mean slope  = −0.82±0.12) and BMC-IV group (Pearson's r = −0.78, mean slope = −0.41±0.19).

## Discussion

The main finding of the present study is that direct intramyocardial injection of BMCs results in improved cell retention, cardiac remodeling and attenuation of cardiac dysfunction. This is consistent with the observation that the IM route was the most efficient for cardiac cell retention and that the other routes of administration, IV, LV or LV^+^, were surprisingly inefficient in this respect under the present experimental conditions. Moreover, regardless of the route of administration, there was no time effect on cell retention for cells delivered 1 to 3 days post-MI, whereas for the IM injection the cell retention was significantly higher when performed 7 versus 1, 2, or 3 days post-MI. Our data also showed that cardiac cell retention can be further improved by an injectable fibrin scaffold in the first 3 days post-MI, even though the morphometric and function parameters evaluated 4 weeks later tended to be similar with or without the injectable polymer scaffold.

The most suitable timing for therapy has been object of previous investigation, however, the studies analyzed and compared the efficacy of cell injections performed immediately after infarction (an acute scenario) with those performed several days after infarction (a more chronic scenario) [22], [23]. Instead, we evaluated cell accumulation at the heart following the injection of cells at 1, 2, 3 or 7 days post-MI; the time window during which the local availability of key cytokines occurs [4]–[6], [24]. We found no evidence for a significant effect of timing on cardiac cell accumulation in the first 3 days post-MI, conversely, for the IM route, the retention was higher at 7 days compared to 1, 2 or 3 days post-MI. Interestingly, these data are in general agreement with a recent clinical finding where optimal reperfusion therapy associated with intracoronary BMC infusion resulted in small but significant improvement in absolute left ventricle ejection fraction when cells were infused from days 5–7 post-reperfusion, but not with cells injected within 4 days post-reperfusion [25]. It appears that cell injections performed one week after cardiac ischemia display better survival in the myocardium and better potential to prevent cardiomyocyte apoptosis and to preserve cardiac function [36–28] since the inflammatory response is reduced while the neoformation of the tissue scar is still in progress [29]–[31].

Remarkably, the data of the present study provide evidence for widespread distribution of transplanted cells and limited cardiac accumulation, regardless of the delivery route employed. The IM route was the most efficient in terms of cardiac cell retention but still relatively unsatisfactory considering that the cells are transplanted directly into the stroma of the target organ. This is in general agreement with previous reports showing that direct intramyocardial cell delivery is the most efficient for cardiac cell retention [32], but still represents a small percentage of the injected cells regardless of the route used [16], [23]. Hofmann et al [33] found the labeled CD34-enriched cell showed superior cardiac cell retention compared to unselected bone marrow cells after intracoronary injection. Several mechanisms of cell loss may influence this outcome including technical problems associated with the delivery, the passive clearance from the site of injection through the vascular and lymph systems, and cell death [22], [34]–[37], which may also explain the greatest variability of the results observed in the IM route [38].

To circumvent some of these limitations and to optimize retention and survival of transplanted cells acutely post-MI, we tested fibrin polymer that is a biodegradable structural matrix important in wound healing, improves transplanted cell survival, induces angiogenesis, reduces infarct expansion, and preserves cardiac function after a myocardial infarction [7]–[9]. However, to date, no study has specifically evaluated the cell retention capacity of this injectable scaffold. Here, we demonstrated that the fibrin polymer increases 2.5 times the amount of transplanted cells remaining at the myocardium 24 hours after an IM injection performed 3 days post infarction. Moreover, similar response was also observed with other more homogeneous cell types, cardiac fibroblasts and adipose-derived stem cells, indicating that this strategy may also be used with different cell types. Surprisingly, the higher fibrin-induced cardiac cell retention resulted in similar cardiac morphometric and functional benefits as observed with the cells injected without the biopolymer. It is tempting to speculate that the unequivocal cell therapy response may be masked early on by the combined and potentiated effect with the endogenous compensatory mechanisms elicited by the acute myocardial ischemia and one cannot exclude the possibility that the added value of a higher number of cells may be measurable beyond 4 weeks post infarction and the cell treatment. This is supported by evidence of long-term improvement of cardiac function associated with stem cell treatments in animal models of cardiac ischemia [39]. Furthermore, the combined effect of the cell therapy and the fibrin polymer scaffold may also influence the cellular matrix. We observed an enhancement scar thickness that may affect LV geometry and improve cardiac function as suggested by the negative correlation observed between scar thickness and LV perimeter (figure 4D). This is consistent with the idea that halting LV elliptical to spherical progression and development of a greater LV volume is beneficial since LV volume has high predictive value for worsening survival post infarction [40].

We failed to demonstrate any improvement on cardiac function in animals that receive BMC by the IV route while others have succeeded [41]–[43]. This may be explained by differences in the animal model used, the cell number injected and the cell type. In the Wolf's study, pigs that received increasing doses of bone marrow mesenchymal cells (until 10×10^6^ cells/kg) displayed improvements in cardiac function only with the higher cell doses. Similar findings were reported by Yoshitaka et al using a 10 times higher number of cells than the reported in our study. Additionally, the animals were weekly injected with cells optimizing the cell availability.

Special care was taken to correct the radioactivity values in the cardiac tissue according to the rate of ^99m^Tc leakage from labeled BMCs. We validated the correction and showed that the leaked ^99m^Tc retention was negligible since it was virtually flushed from the heart and, thus, the radioactivity values can likely be attributed to labeled BMCs. Furthermore, the validation experiment was performed by cardiac injection of the precise volume of supernatant, where a corresponding number of labeled BMCs were incubated for 24 hours. This is important since chromatographic examinations of the supernatant revealed that the leaked ^99m^Tc is a mixture of radiochemical species, which is different from that used to label the cells (data not shown). Moreover, it has been previously demonstrated that the IV injection of ^99m^Tc-HMPAO, which is the complex that labels the cells, does not result in any significant myocardium uptake [44]. At last we confirmed that ^99m^Tc leakage from BMC is not associated with cell death. This radioactive complex can be used for label different cell types including progenitors and undifferentiated cells [45].

It is also important to emphasize that: 1. measurement of radioactive emissions from the entire organ is far more reliable than direct cell enumeration by histological analyses of a limited number of heart sections; and 2. we performed direct radioactive tissue counts and not indirect imaging assessment so the effect of analyzing the samples 24 h later (approximately 4 isotopic ^99m^Tc half-lives), does not limit our measurements. Consequently, we believe that the use of radioactivity instead of direct enumeration techniques represents an improvement on the estimation of cardiac cell retention delivered by different routes.

Taken together, our data show a clear pattern of cell body distribution post-MI using four different routes. There is no time effect for cardiac cell retention when cells are administered 1 to 3 days post-MI, but for the IM route, injection at 7 days is more efficient than injections at 1, 2, or 3 days post MI. The retention of cells by the IM administration 3 days post-MI can be significantly improved by an injectable fibrin scaffold, even though the cardiac morphometric and functional parameters evaluated 4 weeks later were similar. Finally, we provided evidence that direct BMC intramyocardial injection is associated with more efficient cell retention than the IV, LV and LV^+^ routes and it lead to a significant attenuation of cardiac dysfunction associated with myocardial infarction.

## Materials and Methods

### Ethics Statement and Animal Ischemia Model

The experimental procedures followed the institutional guidelines for care and use of laboratory animals and were approved by the Institutional Review Board of the University of São Paulo Medical School, Brazil (#527/04). Ten-week-old male inbred Lewis rats were kept on a rat chow diet and water *ad libitum* and housed under an alternating 12-h light–dark cycle. Experimental myocardial ischemia was produced by ligation of the descending left coronary artery as described before [46]. Briefly, a lateral thoracotomy was performed under anesthesia and the left coronary artery was looped by a single nylon suture (5.0) at approximately 1 mm from its origin and gently tied for 45 min and then released. This procedure produced a clearly demarcated (cyanotic and bulging) area of acute ischemia corresponding to the distribution of the left coronary artery distal to the occlusion. The chest was closed and rats were individually caged during a 24-hour period for recovery.

### Cell isolation and radiolabeling

Under sterile conditions, the femur and tibia of ten-week-old male Lewis rats were excised, and connective tissue was removed. Bone marrow (BM) plugs were extracted from the bones by flushing their cavities with Dulbecco's modified Eagle's medium (DMEM). The resulting BM suspension was carefully minced by passing it through subsequent pipettes of decreasing sizes. The red blood cells were removed by density-gradient centrifugation at 829 *g* for 30 minutes after adding an equal volume of Ficoll-Paque™ Plus (Amersham Bio Sciences AB, Uppsala, Sweden) solution to the BM suspension. Following centrifugation, the low density fraction, composed of the so-called fresh unfractioned bone marrow cells (BMCs), was collected and rinsed with phosphate-buffered saline (PBS).

Ceretec® lyophilized kit (Amersham) was reconstituted in 2 mL of 0.9% NaCl solution containing 1.48 GBq (40mCi) of sodium pertecnetate (IPEN-CNEN, Brazil). Radiochemical purity of the labeled product [technetium 99m–hexamethylpropylene amine oxime (^99m^Tc-HMPAO)] was determined by ethyl acetate/saline extraction procedure. BMCs were labeled with ^99m^Tc-HMPAO, with a minimum changes according to the fabricant recommendation for labeling leucocytes [47]. Briefly, the suspension of BMCs was centrifuged, supernatant was removed and the pellet was resuspended in 1 mL of ^99m^Tc-HMPAO solution and incubated for 15 min at 37°C. Plasma was then added to interrupt cell tagging and the suspension was centrifuged at 466 *g* for 10 minutes. The supernatant was discarded and the pellet resuspended in PBS. The centrifugation and suspension procedure was repeated. The labeling efficiency was assessed and calculated as the ratio of the activity in ^99m^Tc-BMCs to the total radioactivity (radioactivity in cells plus in discarded supernatant).

To evaluate BMC labeling stability, a suspension of 6×10^6^ cells was incubated for 24 hours and then centrifuged. Radioactivity was measured in the pellet and in the supernatant and the rate of leakage was calculated as labeling efficiency. In order to examine the potentially harmful effects of radiation or chemical components of ^99m^Tc-HMPAO, BMCs viability was determined by the trypan blue dye exclusion test.

### Cell Delivery Methods

One, 2, 3 or 7 days after the myocardial ischemia, the animals were subjected to another surgical procedure for a single injection of BMCs (6×10^6^ cells/100 µL of serum-free DMEM) by one of the following experimental routes: *intravenous route* (IV), injection of the cell suspension into the tail vein; *left ventricular cavity route* (LV), transepicardial injection directly into the cavity of the left ventricle; *left ventricular cavity/intracoronary route* (LV^+^), transepicardial injection into left ventricular cavity with aorta occlusion for 20 seconds in order to mimic an intracoronary infusion; and *intramyocardial route* (IM), transepicardial injection within the infarct border zone of the anterior left ventricular free wall. Organs were harvested for analysis twenty four hours after cell transplantation.

### Nuclear radiometry of harvested organs

The animals were euthanized and the heart, lungs, liver, spleen, kidneys, femur and a sample of blood (approximately 3 mL) were harvested and weighed. The radioactivity of the whole isolated organs was determined in a gamma counter (Automatic Gamma Counter 1480 – Perkin Elmer). The radioactivity values of blood and bones were estimated from the amount of radioactivity in samples of these tissues, considering them as 7% and 10%, respectively, of the mass of the entire animal [48]. The results were expressed as a percentage of total injected radioactivity after correction for radioactive decay.

### Heart uptake of ^99m^Tc metabolites

In order to evaluate heart retention and uptake of ^99m^Tc metabolites leaked from BMCs, 30×10^6^ cells were incubated in 500 µL of culture medium for 24 hours. The cell suspension was then centrifuged and 100 µL of the supernatant were injected in the myocardium of 5 animals, 24 hours after infarction. After another 24 hours, animals were euthanized and the hearts harvested for nuclear radiometry.

### Fibrin polymer

The fibrin polymer was prepared by combining fibrinogen and thrombin at the time of injection. Fibrinogen was obtained by separating the plasma from 50 mL of rat whole blood and then adding 5 mL of 3.8% sodium citrate. Finally, the fibrinogen was isolated using the cryoprecipitation technique [49] and diluted to a final concentration of 316 mg/dL. Human thrombin (Baxter Healthcare, Inc.) was used to catalyze fibrin polymerization.

Cells were resuspended in 80 µl of the fibrinogen solution and, a few seconds before injection in the tissue, 20 µl of thrombin (250 UI/ml) were added to the syringe containing the cell suspension. This combination allowed a suitable time window of 20 seconds to perform the myocardial injection before polymerization.

### Hemodynamic study and pharmacologic stress

Four weeks after myocardial infarction, invasive hemodynamic evaluation was performed on a heated rodent operating table (37°C), under adjusted anesthesia (urethane 1.2 g/kg), and oxygen-enriched mechanic ventilation. Left femoral vein was accessed to supplement anesthesia and drugs or saline administration. Bilateral vagothomy was produced to prevent changes on heart rate as a result of the barorreflex in response to pressure pharmacologic stress. A Millar micro manometer (MikroTip™ 2F, Millar Instruments Inc., Houston, TX, USA) was inserted from the right carotid artery to the LV cavity to access intraventricular pressure. A blood flow ultrasound probe (Transonic Systems Inc. NY, USA) was positioned on the ascending aorta to access stroke volume (excluding coronary flow), through right thoracotomy. Data were acquired by the software Acknowledge for windows (Biopac Systems, CA, USA) to get systolic (LVSP) and end-diastolic LV pressures (LVEDP), rate of LV pressure rise (dP/dt), heart rate (HR), cardiac output (CO) and stroke volume (SV). Stroke work (SW) was estimated offline as a product of stroke volume×developed pressure (LVSP – LVEDP)×constant 0.0136.

Hemodynamic parameters were determined under basal condition and after an afterload stress induced by sudden pressure-overload with a vasoconstrictive phenylephrine bolus injection (PHE, 25–75 µg/kg body wt) through the left femoral vein. PHE doses were adjusted for each animal to produce comparable blood pressure increases (60 to 80% of baseline).

### Morphometric Analysis

At the end of experimental procedures, hearts were fixed in 10% formalin for 24 hours, embedded in paraffin and cut into 5 µm sections that were mounted onto slides and stained with *Picrossirius Red* for measurement of collagen scar, left ventricular cavity perimeter (LV cavity) and thickness scar. This procedure was performed in samples obtained from middle and apical transversal segments of the heart. Images of slices were obtained by a software system of image acquisition (NIS - Elements AR 2.30) and the measurements by UTHSCSA ImageToll® software. Infarct size was quantified by the percentage of left ventricular perimeter containing scar tissue and the mean of values from segments was utilized; LV cavity and thickness scar were expressed in millimeters (mm).

### Statistical Analysis

Differences among groups were compared by two way ANOVA test for 3 or more groups and one way ANOVA to compare 2 groups. The Bonferroni Post hoc analysis was performed when the *P* value was lower than 0.05 with a compliance interval of 95%. Results are expressed as means±standard errors of the mean (SEM).

## Supporting Information

Methods S1 (0.04 MB DOC)

Table S1 Cardiac Retention of 99mTc- BMCs 1, 2, 3 or 7 days after myocardial infarction according to the route for cell injection
(0.03 MB DOC)

Figure S1 Transverse sections of hearts from animals that received IM injections of cardiac fibroblasts. A and C show a heart that received cells in standard vehicle, while B and D show a heart that received cells mixed with fibrin. In A and B the heart sections were assessed by β-galactosidase assay (green). In C and D, sections were observed under fluorescence microscopy for nuclei visualization by DAPI staining (blue).
(0.37 MB TIF)

Figure S2 Survival of transplanted cells for 30 days. Heart sections from animals that received 30 days earlier IM injection of ASC (adipose stem cells) labeled with CM-DiI (shown in red and indicated by arrows) mixed with fibrin (A and B, 200×; C and D, 400× magnification, respectively). Nuclei are shown in blue (DAPI staining).
(0.60 MB TIF)
