# Supplementary material for: Cell Therapy Attenuates Cardiac Dysfunction Post Myocardial Infarction: Effect of Timing, Routes of Injection and a Fibrin Scaffold
Source: PLoS One. 2009 Jun 23;4(6):e6005. doi: 10.1371/journal.pone.0006005 (PMC2695782; doi:10.1371/journal.pone.0006005)
Supplement: Methods S1 — (0.04 MB DOC) [file pone.0006005.s001.doc]

**SUPPLEMENTARY METHODS S1**

**Cardiac fibroblasts and adipose-derived stem cells isolation**

Cardiac fibroblasts were isolated from male Lewis rats. Hearts were removed, minced and dissociated with collagenase II (200U/mL, Sigma Chemical Co., Milwaukee, WI, USA) at 37°C for 20 minutes under agitation. The enzyme digestion was arrested by adding 1mL of FBS (fetal bovine serum, Gibco) and the product was centrifuged at 298*g* for 5 minutes. The supernatant was discarded and the cells resuspended in Dulbecco’s modified Eagle’s medium (DMEM) with 15% of FBS (vol./vol.), and 1% (vol./vol.) penicillin–streptomycin (1000UI/mL–1000Ag/mL; GIBCO BRL, Gaithersburg). Cells were plated and maintained in a humid atmosphere at 37°C with 5% of CO2. Cardiac fibroblasts were used after passage 3.

For adipose-derived stem cells (ASCs), inguinal adipose tissue was removed from male Lewis rats and cut in small fragments. Then, the fragments were dissociated with 0.075% collagenase (type IA; Sigma-Aldrich, St. Louis, MO) in DMEM low glucose (DMEM L) with 20mg/g of tissue of Albumin Bovine (BSA, Sigma) at 37°C for 40 minutes under agitation. The enzyme activity was arrested with 10mL of FBS. The solution obtained was centrifuged for 10 minutes at 466g and the pellet was resuspended in DMEM L 10% FBS. The cells was platted at 1x10^4^ cells/cm^2^ and used for experiments in passages 3 and 4. Before injection, CM-DiI (C-7000, Molecular Probes Inc., Oregon, U.S.A) was used to label the cells. Heart slices (5μm) were incubated with DAPI (4’6 – Diamine-2-phenylindole dihydrochloride, Sigma) for nuclei identification.

**Cell infection**

One day before cell transplantation, cardiac fibroblasts were infected with 1x10^10^ pfu/mL of replication-deficient adenovirus expressing LacZ under the control of a viral promoter (AdRSVLacZ). On the day of transplantation, cells were harvested, washed extensively in phosphate-buffered saline (PBS) to remove any free viral particles and labeled with DAPI. Finally, 1x10^6^ cells were resuspended in 100 μL of serum-free DMEM or in fibrinogen solution. Cells injections were performed by the intramyocardial route (IM) 24 hours after infarction.

### β-galactosidase assay

In order to identify and visualize the transplanted cells, 24 hours after IM injection the animals were euthanized and their hearts removed and frozen in OCT compound (Tissue-Tek, Sakura Finetek, U.S.A. Inc. Torrance, CA). Slices of 5μm were fixed (formaldehyde 2% and glutaraldehyde 0,2%) for 10 minutes, washed with PBS and treated for 24 hours with a solution containing 0,1% of galactosidase enzyme (x-gal), 5mM of K ferricyanide, 2mM de K ferrocyanide, and 2mM of MgCl_2_ in PBS. Finally, the slices were stained with hematoxilin and eosin.
