# Supplementary material for: Cell Therapy Attenuates Cardiac Dysfunction Post Myocardial Infarction: Effect of Timing, Routes of Injection and a Fibrin Scaffold
Source: PLoS One. 2009 Jun 23;4(6):e6005. doi: 10.1371/journal.pone.0006005 (PMC2695782; doi:10.1371/journal.pone.0006005)
Supplement: Table S1 — Cardiac Retention of 99mTc- BMCs 1, 2, 3 or 7 days after myocardial infarction according to the route for cell injection (0.03 MB DOC) [file pone.0006005.s002.doc]

**Table S1**.Cardiac Retention of ^99m^Tc- BMCs 1, 2, 3 or 7 days after myocardial infarction according to the route for cell injection

| **Route** | **1d** | | **2d** | | **3d** | | **7d** | |
| --- | --- | --- | --- | --- | --- | --- | --- | --- |
|  | **N** | **%** | **N** | **%** | **N** | **%** | **N** | **%** |
| **IV** | 4 | 0.14 (0.02) | 7 | 0.22 (0.05) | 4 | 0.13 (0.02) | 5 | 0.04 (0.00) |
| **LV** | 4 | 0.43 (0.02) | 4 | 0.49 (0.12) | 4 | 0.71 (0.27) | 5 | 0.18 (0.11) |
| **LV^+^** | 4 | 0.62 (0.15) | 5 | 1.28 (0.36) | 4 | 0.53 (0.1) | 4 | 0.32 (0.13) |
| **IM** | 5 | 8.27 (2.04) | 7 | 4.83 (1.51) | 5 | 6.55 (1.98) | 4 | 6.54 (4.36) ^*§^ |

Percentages are presented as mean (± SE). *N* indicates the number of animals in each group. * (*P*<0.01) indicates difference between IM 7d and IM 1, 2 and 3d. ^§^ (*P*<0.001) indicates difference between IM and IV, LV and LV^+^ routes.
