# Supplementary figures and images for: Cell Therapy Attenuates Cardiac Dysfunction Post Myocardial Infarction: Effect of Timing, Routes of Injection and a Fibrin Scaffold
Source: PLoS One. 2009 Jun 23;4(6):e6005. doi: 10.1371/journal.pone.0006005 (PMC2695782; doi:10.1371/journal.pone.0006005)

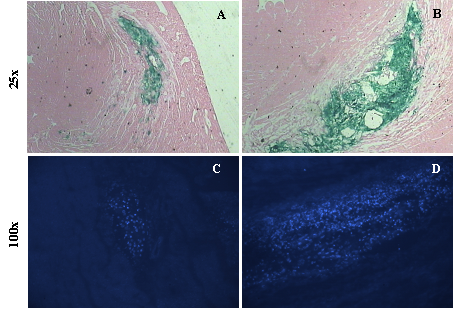

Supplement: Figure S1 — Transverse sections of hearts from animals that received IM injections of cardiac fibroblasts. A and C show a heart that received cells in standard vehicle, while B and D show a heart that received cells mixed with fibrin. In A and B the heart sections were assessed by β-galactosidase assay (green). In C and D, sections were observed under fluorescence microscopy for nuclei visualization by DAPI staining (blue). (0.37 MB TIF) [file pone.0006005.s003.tif]

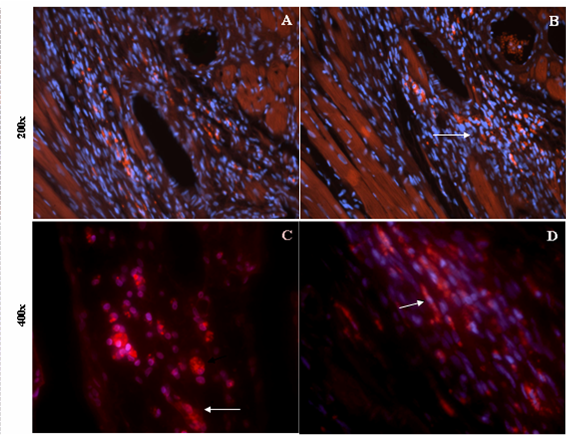

Supplement: Figure S2 — Survival of transplanted cells for 30 days. Heart sections from animals that received 30 days earlier IM injection of ASC (adipose stem cells) labeled with CM-DiI (shown in red and indicated by arrows) mixed with fibrin (A and B, 200×; C and D, 400× magnification, respectively). Nuclei are shown in blue (DAPI staining). (0.60 MB TIF) [file pone.0006005.s004.tif]
